# The Impact of Socio-Economic and Home Environmental Factors on Oral Health-Related Quality of Life Among Children Aged 11–14

**DOI:** 10.3390/medicina55110722

**Published:** 2019-10-31

**Authors:** Shahenaz Najjar, Maliha Nasim, Lubna Al-Nasser, Emad Masuadi

**Affiliations:** 1 Arab American University of Palestine, Ramallah 600, Palestine; 2 King Abdullah International Medical Research Center, King Saud Bin Abdulaziz University for Health Sciences, and Ministry of National Guard-Health Affairs (MNG-HA), Riyadh 11426, Saudi Arabia; 3 Department of Epidemiology, Mailman School of Public Health, Columbia University, New York, NY 10032, USA; 4 Department of Medical Education, College of Medicine King Saud bin Abdulaziz University for Health Sciences, Riyadh 11426, Saudi Arabia

**Keywords:** oral health, health-related quality of life, HRQoL, adolescent/children, Saudi Arabia

## Abstract

*Background and Objectives:* Oral diseases are known to negatively impact physical, functional, and emotional well-being, and thus adversely affect quality of life. The aims of the study were (1) to assess the oral health-related quality of life (OHRQoL) and (2) to explore socio-demographic, -economic, and -environmental factors that are associated with OHRQoL among a sample of children aged 11–14 in Saudi Arabia. *Materials and Methods:* A cross-sectional design was used. The Child Perceptions Questionnaire (CPQ)—a self-administered, validated, and standardized questionnaire was used to collect data on OHRQoL in four domains: oral symptoms, functional limitations, and emotional and social well-being. In addition, data were collected on home environment, socioeconomic/demographic characteristics, and oral hygiene practices of participants and their parents or adult guardians. Univariate descriptive statistics, Spearman’s correlation, and Kruskal–Wallis H and Mann–Whitney tests were used. Data were analyzed using SPSS 23 Software. Significance was set at α = 0.05. *Results:* In total, 534 children participated in the study (91% response rate), of which 60% were females. Twenty percent of children described their oral health as “poor” and one in every four children reported that their oral health had at least some effect on their overall well-being. Children who were male, attending public schools, and living with both parents were more likely to report poor OHRQoL. *Conclusions:* A considerable proportion of children aged 11–14 could discern that their oral health had some effect on their overall well-being. The results identified potential predictors of OHRQoL. Disparities in OHRQoL exist among certain sub-populations. Active efforts and local interventions are necessary to improve OHRQoL.

## 1. Introduction

Oral health is a well-known global public health concern. The World Health Organization (WHO) has estimated that 60–90% of all school-age children are affected by oral health diseases most commonly untreated dental caries [1]. Poor oral health is known to “undermine self-image and self-esteem, discourage social interaction, and cause other health problems that lead to chronic stress and depression as well as incur great financial cost” [2]. Oral health-related quality of life (OHRQoL) strives to capture not only the health status but the broader experience of health as perceived by individuals [2,3]. Multidimensional in its approach, OHRQoL is a subjective, self-reported evaluation based on functional, psychosocial, and economic parameters that impact the individuals’ overall quality of life [2]. The Wilson Clearly conceptual model has been applied as a framework for OHRQoL; linking clinical, non-clinical, individual, and environmental characteristics (including socio-economic factors) with health-related quality of life [4]. Measures of OHRQoL are regarded as oral health outcomes and are useful and important in the assessment and evaluation of oral health status; needs assessment, care provision, and effectiveness of different oral health interventions and actions within a population [5,6,7]. Several patient-based assessment instruments have been developed to measure OHRQoL; however, in children, the child perceptions’ questionnaire (CPQ_11–14_) is the most extensively used. The CPQ_11–14_ assesses four domains: oral symptoms, functional limitations, and social and emotional well-being [8]. The 36 item instrument is designed to assess OHRQoL in children aged 11–14 years, and questions ask about the frequency of events in the previous three months.

This age marks the final transition towards permanent dentition. Moreover, the age period of 10–14, also known as early adolescence, is a transformative period (characterized by developmental, cognitive, and risk-taking behavior) that may affect short- and long-term health and quality of life [9].

Low family income [10,11,12,13,14,15], increased number of siblings [9], and ethnicity and race [16,17] have been significantly associated with poor OHRQoL in children [18,19]. In contrast, children with educated parents and more mature mothers in age reported better OHRQoL [10].

So far, the majority of studies investigating the impact of socio-environmental factors on children’s OHRQoL have been conducted in developed countries [10,11,12,17]. Saudi Arabia (SA) has a population exceeding 31.5 million; more than 2.5 million of its population are children aged 11–14 years [20]. To the best of our knowledge, studies in Saudi Arabia (SA) have focused on OHRQoL as an effectiveness measure in relation to specific dental treatments (i.e., orthodontic or dental rehabilitation under general anesthesia) [21,22] or targeted the parental perception of OHRQoL among a specific subpopulation (children with autism) [23]. One review article estimated that prevalence figures for dental disease among children are as high as 94% for 3–7 years and 91% for 12–19 years [24]. Therefore, dental diseases are likely to be a major public health problem affecting children within SA. The problem’s scale and lack of available information regarding oral health status and OHRQoL of children in SA warrants further study. The aims of this study were to assess the OHRQoL among a sample of SA children and to identify the socio-demographic, -economic, and -environmental factors that impact OHRQoL in this group.

## 2. Materials and Methods

A cross-sectional design was used to achieve the aim of this study. Data were collected by means of a self-administered validated standardized questionnaire addressing OHRQoL. Information was also collected on home environment and socioeconomic/demographic characteristics of children and their parents/guardians. The study protocol was approved by the institutional review board office, King Abdullah International Medical Research Center, Saudi Arabia (SA), research protocol: RC16/012/R (January 2016). Eligibility criteria for participants included: (1) children between 11 to 14 years and (2) accompanied by a parent or an adult guardian (sibling). Children on school trips accompanied by teachers were excluded. Our study was conducted during the Al-Janadriyah festival 2016, which is SA’s national festival of heritage and culture. The Al-Janadriyah festival is the largest festival in SA and it attracts people from all around the different regions of the country. Around 6 million guests attended the festival in 2016 with approximately 20% being children aged 10–14, reflecting the high percentage of children this age throughout the entire Saudi population.

Data were collected electronically using tablet devices. Participants were randomly selected using non-probability sampling. After obtaining the informed consent from an accompanying parent/adult guardian and verbal assent from the child, the child was handed a tablet device to complete the survey on their own. Upon completing the child’s section, guardians were handed the tablet and asked to complete questions separately on socio-demographic and -economic characteristics, and their own oral hygiene behavior. Adding to the oral health-related quality of life sections, data were also collected on family structure, number of siblings, gender of the child, and type of school.

Parents or guardians provided data on their age, gender, marital status, and number of children aside from participant and other household members, parental occupation, education, income, home ownership, access to dental care services, utilization of dental services, and dental health coverage. 

To assess OHRQoL, the Arabic version of the 36 item child perceptions questionnaire (CPQ_11–14_) was used. The CPQ_11–14_ was initially developed and validated in Toronto, Canada by Jokovic et al. [25]. The Arabic version of this questionnaire displayed good psychometric properties and was translated and validated by Brown et al. [26]. The higher the CPQ_11–14_ score, the lower (worse/poorer) the quality of life. In addition to the 36 items of the CPQ_11–14_, two questions that self-rate the overall oral health and the effect of oral health on overall participant well-being were asked and measured on a five-point response format (poor to excellent, never to very much, respectively). A further question regarding oral health practices, such as frequency of the child’s daily teeth brushing, was also included. A subject was considered with good overall health if he/she had answered the question with “very good” or “excellent”. Furthermore, a subject was considered without oral health effect on overall well-being if they answered the question with “none” or “little” effect.

The outcome variables in the study were overall OHRQoL (overall CPQ), the four sub-domains (i.e., oral symptoms, functional limitations, emotional well-being, social well-being), the self-rated overall oral health and overall well-being. Assuming that 50% of Saudi children had better oral health-related quality of life (OHRQoL), with a margin of error 5%, and confidence level of 95%, the minimum recommended sample size was 384 adolescents. To accommodate for non-response and invalid questionnaires, 584 adolescents were invited to participate.

The data were analyzed using SPSS 23 software (IBM Corp., Armonk, N.Y., USA). Data normality was tested using the Kolmogorov–Smirnov test. The domains’ scores and overall score deviated significantly from normal distribution; therefore, non-parametric tests were applied. Univariate descriptive statistics were used to summarize the respondents’ characteristics. The Chi square test was used for proportional comparisons of categorical variables. Spearman’s correlation test was used to test the correlation between the age of parents/guardians and the overall OHRQoL score. Mann–Whitney and Kruskal–Wallis H tests were used to compare the median differences of OHRQoL scores and its domains according to different children’s socio-environmental and demographic independent variables, with Bonferroni correction used when needed. Based on the participants’ total scores of CPQ, the median was used as the cut-off point for OHRQoL. Scores less than or equal to the median were considered as “better” and scores more than the median as “worse”. Binary logistic regression was performed to ascertain the effect of demographic, socio-economic, and environmental characteristics on the overall oral health-related quality of life. A test was considered significant if the *p*-value < 0.05.

## 3. Results

### 3.1. Participant Characteristics

Five hundred and eighty-four children and their parents/guardian were invited to take part in the study. A total of 534 children participated (91% response rate). Twenty-five respondents did not fill out at least 75% of the survey’s questions (so they were omitted from the study). Five hundred and nine questionnaires were used for further analyses. Sixty percent of the participants were male children. The mean age of the adolescents was 12.6 years old, while the mean age of their parents/guardians was 42 years old. Almost 60% were accompanied by their father, 33% with their mother, and 7% with their sibling. The majority (70%) of the children’s mothers were unemployed. However, 90% of their fathers were employed, with nearly two-thirds working in the government sector. More details are described in Table 1.

### 3.2. Relationship between Participants’ Characteristics and Overall OHRQoL Scores

The parent/guardians’ age significantly correlated with OHRQoL score. The median OHRQoL reported among females was significantly lower (better) than males. Participants studying at international and private schools reported significantly better median OHRQoL scores compared to those studying at government schools. The median OHRQoL scores of participants whose mothers and fathers working within the healthcare field were significantly better than in other fields. Participants not living with both parents had significantly better OHRQoL scores compared to participants living with both parents. However, the OHRQoL scores among mothers (as a caregiver type) were better than fathers and other guardians. Children or parents/guardians who brushed their teeth more than two times per day had better OHRQoL compared to those who brushed once a day or did not brush at all. More details are reported in Table 1.

### 3.3. Relationship between Participants’ Characteristics on Overall Oral Health and Effect of Oral Health on Overall Well-Being (Single Item Outcomes of Q37 and Q38)

Approximately, three in every four children reported their oral health impact as “none/little effect” on their overall well-being. Participant characteristics that were found to be more prevalent with reporting “none/little effect of oral health on overall well-being include: females; (87% versus 71%, *p*-value < 0.001); participants whose mothers had a secondary school education compared to other groups (84%, *p*-value = 0.023); children who do not live with both parents compared to those who do (95% and 89%, *p*-value = 0.004 and <0.001, respectively), and living with a guardian who brushes their teeth two or more times per day compared to subgroups brushing less than frequently than this (84%, *p*-value = 0.001). More details are described in Table 2.

### 3.4. Relationship between Participants’ Characteristics and the Sub-Domains of OHRQoL

The oral symptoms domain was significantly associated with child brushing frequency. Functional limitations were significantly associated with child gender, region, school type, employment status of the father, living with both parents, and caregiver type. Emotional well-being was significantly associated with the employment status of the father, child brushing frequency, and guardian brushing frequency. Finally, child gender, mothers’ education, school type, employment status of the father, home ownership, living with both parents, sibling numbers, caregiver type, and brushing frequency of the child and the guardian were significantly associated with social well-being (other associations are reported in Table 3).

### 3.5. Multivariate Analysis of OHRQoL and Participants’ Characteristics

A binary step-wise logistic regression model (Table 4) showed that as the parents’ age increased by one year, the odds of reporting better oral health-related quality of life decreased by 2% (OR = 0.98, 95% CI; 0.96–1). Furthermore, factors found to increase the odds of better reporting of OHRQoL included: female gender by 58% (OR = 1.58, 95% CI; 1.07–2.34) and attendance at an international/private school by 50% (OR = 1.50, 95% CI; 1.01–2.23). Moreover, children who did not live with both parents were 2.3 times more likely to report better OHRQoL than those who did (OR = 2.34, 95% CI; 1.12–4.86).

## 4. Discussion

This is the first study in SA to describe the associations between OHRQoL and demographic, socioeconomic, and environmental factors among a general population sample. The results revealed that almost 47% of the participants reported “very good/excellent” overall oral health. One in every four children reported “some/very much” effect of their oral health on their overall well-being. Interestingly, the findings suggest that females, young parents, and children attending international/private school report better OHRQoL. Also, children who do not live with both parents were 2.3 times more likely to report better OHRQoL than those who do.

The trend in reporting “some/more effect” of oral health on general well-being is highlighted by the fact that children’s emotional well-being was the most commonly affected domain in this study. Emotional and social well-being are frequently cited in the literature as being prominent parts of the perceived effect of oral disease and/or dysfunction on OHRQoL [27]. As those children (11–14 years) come under the umbrella of early adolescence, the effect on these domains might reflect the psychological changes that occur in early adolescence, where adolescents become sensitized to cultural norms and ideals, and an increased importance is attached to one’s peers’ perception of oneself [27].

Socioeconomic status (SES) is a complex construct that is composed of many inter-connected variables. Our findings suggest that the effect of SES might be mediated by some variables more than others. For example, and in contrast to other studies, girls reported lower OHRQoL than boys in this study [28]. The mismatch could reflect cultural differences in the perception and expression of oral health between girls and boys or reflect true differences in the oral health status among participants. Contrary to findings of other studies [10,18], in this study, children of young parents reported better OHRQoL. A plausible justification could be that the young parents are more exposed to preventive dental care awareness (e.g., brushing frequency or preventive dental visits) and are therefore more likely to be vigilant for oral health impact on children’s quality of life which affected their children’s perceptions of OHRQoL. This is supported by the significant association between the brushing frequency of the participants’ and their parents/guardians and OHRQoL, as well as with overall oral health.

This study showed that children attending international/private school have better OHRQoL. The comparison of school type (public versus international/private) may suggest affluent families would be more likely to afford preventive dental care and more inclined to send their children to private schools. Similarly, other schools (e.g., private or international schools) might encourage their students to follow more health-enhancing behaviors (e.g., exercise, teeth brushing or restricting candy consumption at school lunch) when compared to public schools. SES may also be implicated in the adoption of behaviors that are more conducive to oral health (i.e., increased oral health literacy and/or preventive dental visits), which are by themselves independent factors that can influence oral health status and OHRQoL.

A surprising finding was that children who live in a household without their parents were 2.3 times more likely to report better OHRQoL than those who live at home with their parents. A possible explanation for this may be that when living with one parent, the parent may be more attentive to their children’s health. Many Saudi families rely upon nannies to take care of many aspects of their children’s well-being (which may also include personal hygiene). Who the caregiver is rather than whether or not the child lives with their parents may better explain the difference that exists in reported OHRQol scores. Our study highlighted that mothers as caregivers increased the likelihood of reporting better OHRQoL scores (*p*-value < 0.001). Having a mother as a caregiver may alter their perception of OHRQoL compared to others whose caregiver is a father or guardian.

### Strengths and Limitations of the Study

This study described the predictors of OHRQoL and the perceived impact of oral health on overall well-being of a population-based sample of children and adolescents. Children and adolescents bear the biggest burden of oral dysfunction. Findings from this sample may help inform public health efforts and policies to improve prevention and curative efforts for this vulnerable group. This study was the first to recruit a population-based sample as opposed to hospital-based sample that conceivably might exhibit more oral disease prevalence and a substantially different OHRQoL profile. However, the setting of a festival made recruiting a probability sample logistically unfeasible thereby limiting the generalizability of our results. Furthermore, oral examinations to investigate how clinical and self-reported measures were correlated among the study participants was not performed. In our study, most of our participants reported coming from households with a high monthly income (54% of participants, reported USD $2665 dollar/month); therefore, our findings may be biased towards individuals who are socioeconomically better off.

## 5. Conclusions

Our findings raise awareness of children’s oral health-related quality of life. Collectively, promoting (preventative) oral health care within schools and communities and linking these services with youth-serving organizations is important to improve children’s oral health and overall well-being. Moreover, attention to boys’ and parental oral health should be taken into consideration when designing interventions to enhance oral health. Further studies are recommended to assess the causal pathways by which socioeconomic factors affect OHRQoL and to identify clinical and public health opportunities to improve OHRQoL.

## Figures and Tables

**Table 1:** Association between participants’ characteristics and overall oral health-related quality of life (OHRQoL) scores.

		*N* (%)	Overall OHRQoL	
			Q2 (Q1–Q3)	*p*-Value
Child gender	Female	202 (40)	23 (16–33)	<0.001 *
	Male	307 (60)	31 (22–41)	
Region	Central	478 (94)	28 (19–38)	0.25
	Other	31 (6)	26 (18–35)	
Mother education	Primary school	153 (30)	28 (20–42)	0.455
	Secondary school	188 (37)	29 (19–36)	
	University	168 (33)	27 (19–36)	
Father education	Primary school	100 (20)	29 (21–40)	0.863
	Secondary school	197 (39)	29 (20–36)	
	University	212 (42)	27 (19–40)	
School type	Governmental school	357 (70)	29 (20–40)	0.028 *
	International and Private	150 (30)	26 (18–35)	
Monthly income **	Less than 5000 SAR	41(8)	25 (18–34)	0.129
	5000–10,000 SAR	190 (37)	29 (21–39)	
	More than 10,000	278 (55)	28 (19–37)	
Employment status mother	Healthcare worker	16 (3)	22 (17–28)	0.035 *
	Government worker	103 (20)	28 (20–37)	
	Works in the private sector	22 (4)	22 (18–28)	
	I own my own business	12 (2)	29 (22–40)	
	I do not work currently	356 (70)	29 (19–39)	
Employment status father	Healthcare worker	27 (5)	21 (13–26)	0.011 *
	Government worker	330 (65)	29 (20–40)	
	Works in the private sector	71 (14)	29 (18–36)	
	I own my own business	28 (6)	28 (16–40)	
	I do not work currently	53 (10)	25 (20–32)	
Home ownership	No	235 (46)	27 (18–36)	0.141
	Yes	273 (54)	29 (20–38)	
Living parents	No	42 (8)	21 (18–29)	0.005 *
	Yes	465 (92)	29 (20–38)	
Sibling numbers	No sibling	29 (6)	24 (17–30)	0.139
	Less than two	24 (5)	30 (22–41)	
	Two or more	456 (90)	28 (19–38)	
Caregiver type	Mother	169 (33)	24 (16–34)	<0.001 *
	Father	304 (60)	30 (21–40)	
	Guardian	36 (7)	28 (18–38)	
Brushing frequency of the child	I do not brush	140 (28)	32 (22–41)	0.002 *
	Once a day	144 (28)	27 (19–40)	
	≥2 times/day	225 (44)	26 (18–35)	
Brushing frequency of the guardian	I do not brush	150 (30)	31 (24–46)	<0.001 *
	Once a day	141 (28)	27 (20–36)	
	≥2 times/day	218 (43)	24 (17–36)	
Dental insurance	No	364 (72)	27 (20–38)	0.776
	Yes	145 (29)	29 (18–36)	
	**Mean**	**Standard Deviation**	***r***	***p*-Value**
Age of child	12.6	1.2	0.12	0.006
Age of parents/guardian	42.2	9.2	0.18	<0.001

Q1 = first quartile, Q2 = median, Q3 = third quartile. *R* = Spearman correlation. *p*-Value generated by Mann–Whitney and Kruskal–Wallis H tests. *p*-Value compares the overall OHRQoL (health-related quality of life). * Significance is declared at an alpha less than 0.05. ** 1 SAR = 0.27 USD.

**Table 2:** Relationship between participants’ characteristics with overall oral health reporting and effect of oral health on overall well-being (single item outcomes of Q37 and Q38).

		Overall Rating of Oral Health (Good/Excellent)		Effect of Oral Health on Overall Well-Being (None/Little)	
		*N* (% **)	*p*-Value	*N* (%)	*p*-Value
Child gender	Female	108 (54)	0.017 *	176 (87)	<0.001 *
	Male	131 (43)		218 (71)	
Region	Central	221 (46)	0.201	371 (78)	0.659
	Other	18 (58)		23 (74)	
Mother’s education	Primary school	69 (45)	0.404	112 (73)	0.023 *
	Secondary school	84 (45)		158 (84)	
	University	86 (51)		124 (74)	
Father’s education	Primary school	43 (43)	0.006 *	76 (76)	0.931
	Secondary school	79 (40)		153 (78)	
	University	117 (55)		165 (78)	
School type	Governmental school	155 (43)	0.020 *	276 (77)	0.996
	International and Private	82 (55)		116 (77)	
Monthly income ***	Less than 5000 SAR	22 (54)	0.153	32 (78)	0.902
	5000–10,000 SAR	79 (42)		145 (76)	
	More than 10,000	138 (50)		217 (78)	
Employment status of mother	Healthcare worker	11 (69)	0.283	14 (88)	0.351
	Government worker	50 (49)		74 (72)	
	Works in the private sector	12 (55)		18 (82)	
	I own my own business	7 (58)		11 (92)	
	I do not work currently	159 (45)		277 (78)	
Employment status of father	Healthcare worker	21 (78)	0.006 *	23 (85)	0.196
	Government worker	157 (48)		247 (75)	
	Works in the private sector	25 (35)		55 (78)	
	I own my own business	12 (43)		22 (79)	
	I do not work currently	24 (45)		47 (89)	
Home ownership	No	123 (52)	0.021 *	188 (80)	0.187
	Yes	115 (42)		205 (75)	
Living parents	No	27 (64)	0.017 *	40 (95)	0.004 *
	Yes	210 (45)		353 (76)	
Sibling numbers	No sibling	16 (55)	0.618	25 (86)	0.493
	Less than two	12 (50)		18 (75)	
	Two or more	211 (46)		351 (77)	
Caregiver type	Mother	84 (50)	0.183	150 (89)	<0.001 *
	Father	134 (44)		214 (70)	
	Guardian	21(58)		30 (83)	
Brushing frequency of the child	I do not brush	45 (32)	0.000 *	103 (74)	0.069
	Once a day	65 (45)		106 (74)	
	≥2 times/day	129 (57)		185 (82)	
Brushing frequency of the guardian	I do not brush	58 (39)	0.044 *	101 (67)	0.001 *
	Once a day	74 (53)		109 (77)	
	≥2 times/day	107 (49)		184 (84)	
Dental insurance	No	178 (49)	0.163	276 (76)	0.176
	Yes	61 (42)		118 (81)	

Values determined via chi-square test. * Significance is declared at an alpha less than 0.05. ** This is the % of good or excellent scores. *** 1 SAR = 0.27 USD.

**Table 3:** Participants’ characteristics and sub-domains of OHRQoL.

		Oral Symptoms (Out of 24)		Functional Limitation (Out of 36)		Emotional Well-Being (Out of 36)		Social Well-Being (Out of 48)	
		Q2 (Q1–Q3)	*p*-Value	Q2 (Q1–Q3)	*p*-Value	Q2 (Q1–Q3)	*p*-Value	Q2 (Q1–Q3)	*p*-Value
Child gender	Female	5 (2–8)	0.063	5 (2–8)	<0.001 *	8 (4–12)	0.147	6 (2–9)	<0.001 *
	Male	6 (3–8)		6 (4–9)		9 (6–13)		9 (5–14)	
Region	Central	6 (3–8)	0.053	6 (3–9)	0.046 *	8 (5–12)	0.891	7 (3–12)	0.915
	Other	4 (2–5)		4 (2–7)		9 (4–11)		6 (4–13)	
Mother’s education	Primary school	5 (3–8)	0.961	5 (3–9)	0.931	8 (5–12)	0.859	9 (4–15)	0.015 *
	Secondary school	6 (3–8)		6 (3–9)		8 (5–13)		6 (4–11)	
	University	6 (3–8)		6 (4–8)		9 (6–12)		6 (3–12)	
Father’s education	Primary school	5 (3–8)	0.63	6 (3–10)	0.745	7 (5–12)	0.119	8 (4–14)	0.376
	Secondary school	5 (4–8)		5 (3–8)		9 (5–12)		7 (3–12)	
	University	6 (3–8)		6 (3–9)		9 (6–13)		6 (3–12)	
School type	Governmental school	5 (3–8)	0.8	6 (3–9)	0.018 *	9 (5–12)	0.443	8 (4–13)	0.012 *
	International and Private	6 (2–8)		5 (2–8)		8 (5–12)		6 (3–12)	
Monthly income **	Less than 5000 SAR	4 (2–8)	0.122	4 (2–12)	0.274	8 (5–11)	0.443	6 (4–9)	0.418
	5000–10,000 SAR	6 (3–8)		6 (4–9)		9 (5–12)		7 (4–13)	
	More than 10,000	5 (3–8)		6 (3–8)		9 (6–13)		7 (3–12)	
Employment status of mother	Healthcare worker	5 (3–8)	0.207	5 (3–5)	0.053	8 (4–11)	0.216	5 (3–8)	0.29
	Government worker	6 (3–8)		6 (4–8)		9 (6–12)		8 (3–11)	
	Works in the private sector	4 (2–6)		5 (2–7)		7 (4–8)		6 (3–10)	
	I own my own business	4 (2–8)		9 (4–14)		10 (4–14)		8 (2–15)	
	I do not work currently	6 (3–8)		6 (3–9)		9 (5–12)		7 (4–13)	
Employment status of father	Healthcare worker	4 (2–7)	0.738	4 (1–6)	0.048 *	7 (2–9)	0.002 *	5 (3–7)	0.002 *
	Government worker	6 (4–8)		6 (3–9)		8 (5–13)		8 (4–14)	
	Works in the private sector	6 (3–9)		5 (2–8)		10 (7–12)		7 (3–12)	
	I own my own business	5 (2–8)		7 (3–10)		5 (3–9)		8 (1–14)	
	I do not work currently	6 (3–8)		5 (4–9)		10 (6–12)		4 (2–9)	
Home ownership	No	6 (3–9)	0.323	6 (3–9)	0.475	8 (4–13)	0.474	6 (3–11)	0.001 *
	Yes	5 (3–8)		6 (4–9)		9 (6–12)		8 (4–13)	
Living parents	No	6 (2–8)	0.999	5 (2–5)	0.006 *	8 (6–9)	0.294	5 (2–7)	0.001 *
	Yes	5 (3–8)		6 (3–9)		9 (5–12)		7 (4–13)	
Sibling numbers	No sibling	5 (4–7)	0.78	5 (2–7)	0.354	7 (5–11)	0.077	3 (2–9)	0.036 *
	Less than two	6 (4–8)		6 (4–10)		11 (8–15)		6 (4–13)	
	Two or more	6 (3–8)		6 (3–9)		9 (5–12)		7 (4–13)	
Caregiver type	Mother	6 (3–9)	0.298	5 (3–8)	0.006 *	8 (5–12)	0.374	5 (2–9)	<0.001 *
	Father	5 (3–8)		6 (4–9)		9 (6–13)		9 (5–14)	
	Guardian	6 (4–11)		5 (2–8)		10 (6–12)		6 (3–8)	
Brushing frequency of the child	I do not brush	6 (4–9)	0.003 *	6 (4–9)	0.146	9 (6–13)	0.024 *	9 (6–13)	0.001 *
	Once a day	6 (4–8)		6 (3–9)		8 (5–11)		6 (3–14)	
	≥2 times/day	5 (2–8)		5 (3–8)		8 (5–12)		6 (3–10)	
Brushing frequency of the guardian	I do not brush	6 (3–8)	0.508	6 (4–10)	0.052	9 (6–14)	0.004 *	10 (6–16)	<0.001 *
	Once a day	5 (2–8)		6 (4–9)		8 (6–12)		6 (4–12)	
	≥2 times/day	6 (3–8)		5 (3–8)		8 (4–12)		6 (3–10)	
Dental insurance	No	6 (3–8)	0.276	6 (3–9)	0.824	8 (5–12)	0.356	7 (4–13)	0.406
	Yes	5 (2–8)		6 (4–8)		9 (6–13)		7 (3–11)	

Q1 = first quartile, Q2 = median, Q3 = third quartile. Mann–Whitney and Kruskal–Wallis H tests were used. * Significance is declared at an alpha less than 0.05. ** 1 SAR = 0.27 USD.

**Table 4:** Multivariate analysis of OHRQoL and participants’ characteristics.

		*p*-Value	OR	95% CI for OR	
				Lower	Upper
Parents/Guardians’ age		0.048	0.98	0.96	1.00
Gender	Female	0.022	1.58	1.07	2.34
	Male *		1.00		
School type	International and Private	0.044	1.50	1.01	2.23
	Governmental school *		1.00		
Living with parents	No	0.023	2.34	1.12	4.86
	Yes *		1.00		

Logistic regression tests were used. OR = Odds Ratio. * Reference group.

## References

[1] Pitts N., Zero D. Caries Prevention Partnership Making Prevention a Priority a Summary of the Current Evidence and the Key Issues in Controlling this Preventable Disease. http://www.fdiworlddental.org/sites/default/files/media/documents/2016-fdi_cpp-white_paper.pdf.

[2] US Department of Health and Human Services (2000). Oral Health in America: A Report of the Surgeon General Executive Summary. https://www.nidcr.nih.gov/DataStatistics/SurgeonGeneral/Documents/hck1ocv.@www.surgeon.fullrpt.pdf.

[3] Locker D., Allen F. (2007). What do measures of ‘oral health-related quality of life’ measure?. Community Dent. Oral Epidemiol..

[4] Santos C.M., Celeste R.K., Hilgert J.B., Hugo F.N. (2015). Testing the applicability of a model of oral health-related quality of life. Cad. Saude Publica.

[5] Sischo L., Broder H. (2011). Oral health-related quality of life: What, why, how, and future implications. J. Dent. Res..

[6] Pelo S., Gasparini G., Garagiola U., Cordaro M., Di Nardo F., Staderini E., Patini R., De Angelis P., D’Amato G., Saponaro G. (2017). Surgery-first orthognathic approach vs traditional orthognathic approach: Oral health-related quality of life assessed with 2 questionnaires. Am. J. Orthod. Dentofac. Orthop..

[7] Pelo S., Saponaro G., Patini R., Staderini E., Giordano A., Gasparini G., Garagiola U., Azzuni C., Cordaro M., Foresta E. (2017). Risks in surgery-first orthognathic approach: Complications of segmental osteotomies of the jaws. A systematic review. Eur. Rev. Med. Pharmacol. Sci..

[8] Gilchrist F., Rodd H., Deery C., Marshman Z. (2014). Assessment of the quality of measures of child oral health-related quality of life. BMC Oral Heal..

[9] Clemente R.J., Santelli J.S., Crosby R.A. (2009). Adolescent Health: Understanding and Preventing Risk Behaviors.

[10] Kumar S., Kroon J., Lalloo R. (2014). A systematic review of the impact of parental socio-economic status and home environment characteristics on children’s oral health related quality of life. Heal. Qual. Life Outcomes.

[11] Paula J.S., Leite I.C., Almeida A.B., Ambrosano G.M., Pereira A.C., Mialhe F.L. (2012). The influence of oral health conditions, socioeconomic status and home environment factors on schoolchildren’s self-perception of quality of life. Heal. Qual. Life Outcomes.

[12] de Paula J.S., Leite I.C.G., de Almeida A.B., Ambrosano G.M.B., Mialhe F.L. (2013). The impact of socioenvironmental characteristics on domains of oral health-related quality of life in Brazilian schoolchildren. BMC Oral Health.

[13] Varenne B., Fournet F., Cadot E., Msellati P., Ouedraogo H., Meyer P., Cornu J.-F., Salem G., Petersen P. (2011). Environnement familial et disparités de santé dentaire des enfants en milieu urbain au Burkina Faso. Rev. d’Épidémiol. Santé Publique.

[14] Vargas-Ferreira F., Piovesan C., Praetzel J., Mendes F., Allison P., Ardenghi T. (2010). Tooth Erosion with Low Severity Does Not Impact Child Oral Health-Related Quality of Life. Caries Res..

[15] Piovesan C., Antunes J.L.F., Guedes R.S., Ardenghi T.M. (2010). Impact of socioeconomic and clinical factors on child oral health-related quality of life (COHRQoL). Qual. Life Res..

[16] Nanayakkara V., Renzaho A., Oldenburg B., Ekanayake L. (2013). Ethnic and socio-economic disparities in oral health outcomes and quality of life among Sri Lankan preschoolers: A cross-sectional study. Int. J. Equity Heal..

[17] Emmanuelli B., Kucner Â.A., Ostapiuck M., Tomazoni F., Agostini B.A., Ardenghi T.M. (2015). Racial Differences in Oral Health-Related Quality of Life: A Multilevel Analysis in Brazilian Children. Braz. Dent. J..

[18] Kumar S., Tadakamadla J., Kroon J., Johnson N.W. (2016). Impact of parent-related factors on dental caries in the permanent dentition of 6–12-year-old children: A systematic review. J. Dent..

[19] Bozorgmehr E., Hajizamani A., Mohammadi T.M. (2013). Oral Health Behavior of Parents as a Predictor of Oral Health Status of Their Children. ISRN Dent..

[20] US Census Bureau (2017). International Programs. International Data Base. https://www.census.gov/data-tools/demo/idb/region.php?T=10&RT=0&A=both&Y=2016&C=SA&R=.

[21] Baghdadi Z.D. (2014). Effects of Dental Rehabilitation under General Anesthesia on Children’s Oral Health-Related Quality of Life Using Proxy Short Versions of OHRQoL Instruments. Sci. World J..

[22] Hassan A.H., Hassan M.H., I Linjawi A. (2014). Association of orthodontic treatment needs and oral health-related quality of life in Saudi children seeking orthodontic treatment. Patient Prefer. Adherence.

[23] Pani S.C., Mubaraki S.A., Ahmed Y.T., AlTurki R.Y., Almahfouz S.F. (2013). Parental perceptions of the oral health-related quality of life of autistic children in Saudi Arabia. Spec. Care Dent..

[24] Al-Ansari A. (2014). Prevalence, severity, and secular trends of dental caries among various saudi populations: A literature review. Saudi J. Med. Med Sci..

[25] Jokovic A., Locker D., Stephens M., Kenny D., Tompson B., Guyatt G. (2002). Validity and reliability of a questionnaire for measuring child oral-health-related quality of life. J. Dent. Res..

[26] Brown A., Al-Khayal Z., Al-Khayal Z. (2006). Validity and reliability of the Arabic translation of the child oral-health-related quality of life questionnaire (CPQ11?14) in Saudi Arabia. Int. J. Paediatr. Dent..

[27] Barbosa T., Gavião M. (2008). Oral health-related quality of life in children: Part I. How well do children know themselves? A systematic review. Int. J. Dent. Hyg..

[28] Page L.F., Jokovic A., Locker D., Thomson W.M. (2005). Validation of the Child Perceptions Questionnaire (CPQ 11–14). J. Dent. Res..

